# Hemolysis and hyperhomocysteinemia caused by cobalamin deficiency: three case reports and review of the literature

**DOI:** 10.1186/1756-8722-1-26

**Published:** 2008-12-18

**Authors:** Utkarsh Acharya, Jen-Tzer Gau, William Horvath, Paolo Ventura, Chung-Tsen Hsueh, Wayne Carlsen

**Affiliations:** 1 Department of Geriatric Medicine, Ohio University College of Osteopathic Medicine, Athens, OH, USA; 2 Department of Internal Medicine, University of Toledo College of Medicine, Toledo, OH, USA; 3 Department of Medicines and Medical Specialties, University of Modena and Reggio Emilia, Modena, Italy; 4 Division of Hematology-Oncology, Department of Internal Medicine, Loma Linda University, Loma Linda, CA, USA

## Abstract

Concurrent hemolysis in patients with vitamin B12 deficiency is a well-recognized phenomenon and has been attributed to intramedullary destruction of erythrocytes (ineffective erythropoiesis). Recent studies revealed that homocysteine increased the risk of hemolysis in vitamin B12 deficiency in vitro and there is a high frequency (30%) of vitamin B12 deficiency in asymptomatic patients with homozygous methylene tetrahydrofolate reductase (MTHFR) C677T mutation, a known cause of hyperhomocysteinemia. Here we report three patients with MTHFR mutations and vitamin B12 deficiency presenting with hemolytic anemia and severely elevated homocysteine levels. Patients demonstrated complete resolution of hemolysis with simultaneous normalization of serum homocysteine levels after vitamin B12 treatments. We reviewed pertinent literature, and hypothesized that hemolytic anemia may be more prevalent in patients who have a coexisting MTHFR gene mutation and vitamin B12 deficiency possibly related to severely elevated homocysteine levels. The hemolysis in these cases occurred predominantly in peripheral blood likely due to the combined effects of structurally defective erythrocytes and homocysteine-induced endothelial damage with microangiopathy.

## Background

Hematological consequences of vitamin B12 (cobalamin) deficiency can be severe. It was estimated that 10% of the patients had life threatening conditions such as symptomatic pancytopenia, "pseudo" thrombotic microangiopathy, and hemolytic anemia [1]. Concurrent hemolysis in patients with vitamin B12 deficiency has been attributed to intramedullary destruction of red blood cells (ineffective erythropoiesis) [2]. However, studies revealed that homocysteine accumulation due to vitamin B12 and folate deficiency increased hemolysis in vitro [3,4]. A recent study further demonstrated a high frequency (30%) of vitamin B12 deficiency among 67 asymptomatic patients with homozygous methylene tetrahydrofolate reductase (MTHFR) C677T mutation, a cause of hyperhomocysteinemia [5]. In this case report, we identified three cases of vitamin B12 deficiency and hemolytic anemia associated with severe hyperhomocysteinemia and MTHFR gene mutations. Resolution of hemolysis and normalization of serum homocysteine levels were noted after vitamin B12 treatments. We hypothesize that high homocysteine levels may be an important contributor leading to further hemolysis that is often seen in patients with vitamin B12 deficiency.

## Case presentation

### Case 1

**Figure 1:**
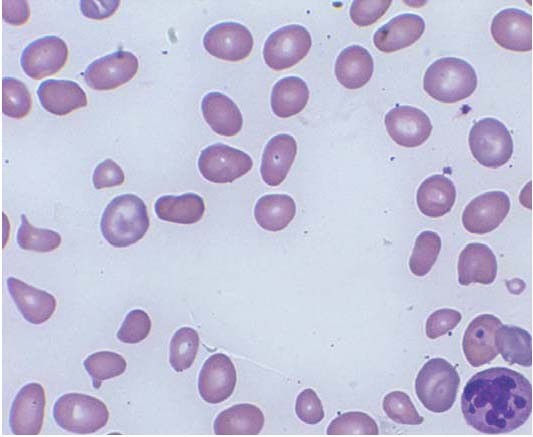
Peripheral smear of case 1 demonstrated schistocytes and a hypersegmented neutrophil.

A 55 year-old white female with a history of hypothyroidism and pernicious anemia, had lost follow-up for 10 years. She presented with lethargy, confusion, exertional dyspnea, difficulty with ambulation, and cold intolerance. Abnormal physical findings included vitiligo, moderate lower extremity edema and a mid-systolic click. Laboratory findings revealed a hemoglobin (Hb) of 5.0 g/dL (normal 12–16) with a mean corpuscular volume (MCV) of 134 fL (normal 80–100); white cell count (WBC) 3,100/mm^3 ^(normal 3,500–11,000); platelet 123,000/mm^3 ^(normal 140,000–450,000); and reticulocyte count 6.3% (normal 0.5–2). Further study revealed vitamin B12 level 167 pg/mL (normal 211–946), homocysteine level 62.4 μmol/L (normal 5.0–13.9), methylmalonic acid level 13.53 μmol/L (normal 0 – 0.40), haptoglobin levels < 6 mg/dL (normal 16–200) and lactate dehydrogenase (LDH) 3152 U/L (normal 100–190). Thyroid studies revealed a thyroid-stimulating hormone (TSH) of 8.26 mlU/L (normal 0.37 – 4.42) with a thyroxin level of 0.71 ng/dL (normal 0.75 – 2.00). Other chemistry studies, including bilirubin levels, were normal. She was heterozygous for methylene tetrahydrofolate reductase (MTHFR) A1298C mutation. Peripheral smear was remarkable for schistocytes and hypersegmented neutrophils (Figure 1).

Patient was transfused with 2 units of packed red blood cells (RBC) and initiated on intramuscular vitamin B12 injections and daily levothyroxine supplement. Six weeks later the Hb was 12.1 g/dL, MCV at 91.2 fL, homocysteine level 14.6 μmol/L, TSH 4.27 and free T4 1.08 with resolved WBC and platelet counts.

### Case 2

A 58 year-old white male with a history of essential hypertension and tobacco use was admitted with complaints of progressively increasing fatigue over the past three to four months. The patient denied hematochezia, hemoptysis, or hematuria. However, the patient did report slight paresthesias in both soles, without significant alterations of reflexes. Further history revealed a relatively recent change in patient's dietary habits as he had adopted a strict vegetarian diet over the past fifteen months due to personal experiences and convictions. His only medication was rimipril.

**Figure 2:**
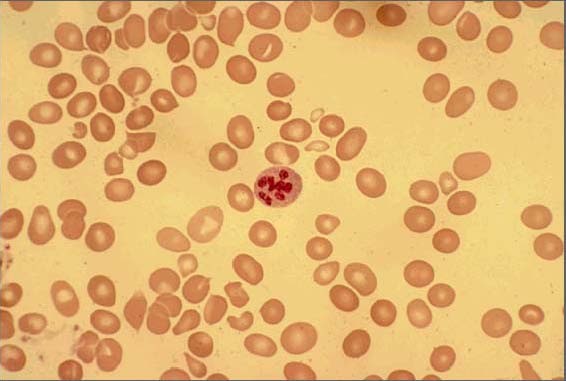
Peripheral smear of case 2 demonstrated macrocytosis with anisopoikilocytosis and one hypersegmented neutrophil/granulocyte with schistocytes.

**Figure 3:**
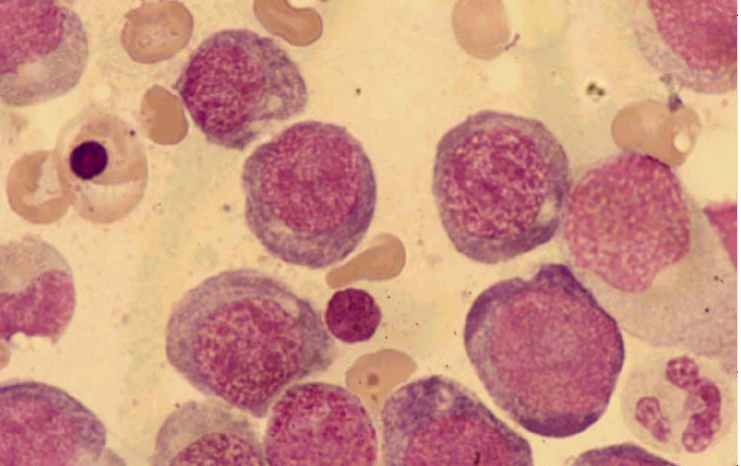
Bone morrow of case 2 demonstrated macromegaloblastic erythropoiesis.

Positive physical findings at the time of admission included a slight conjunctival jaundice with pale skin and weakness in the limbs. The patient was found to have a macrocytic anemia with a slight increase in serum bilirubin levels (2.1 mg/dL) in a screening blood test two weeks prior to admission. Blood count on admission showed a WBC 3,400/mm^3^, Hb 7.7 g/dL (MCV 115 fl), and platelet 99,000/mm^3^. Serum vitamin B12 level was 100 pg/mL, whereas serum folate level, iron study, and thyroid function tests were within normal range. Reticulocyte count resulted 6% (normal range 2–6). Chemistry results were remarkable for serum bilirubin 2.3 mg/dL (1.9 mg/dL as unconjugated), elevated LDH (788 U/L) and serum haptoglobin < 7 mg/dL. Plasma homocysteine level was significantly increased at 88.8 μmol/L with an elevated methylmalonic acid level at 12.1 μmol/L. Serum creatinine, direct and indirect Coombs tests, and glucose-6-phosphate dehydrogenase activity in red blood cells were all normal. The patient resulted homozygous for MTHFR C677T mutation. Peripheral blood smear and marrow biopsy revealed a megaloblastic anemia with megaloblastic erythroid hyperplasia with granulopoiesis and megakaryocytosis (Figures 2 and 3).

During the one-week hospital stay, the patient was transfused with 1 unit of packed RBC and started on intramuscular injections of cobalamin and oral supplementation of folic acid. Meat was re-introduced in the patient's diet. The patient underwent an esophago-gastroduodenoscopy with gastric biopsy which exhibited evidence of atrophic gastritis. Studies for antibodies to intrinsic factor and gastric parietal cells were negative.

The patient was completely asymptomatic 4 months later with the following blood test results: WBC 5,400/mm^3^, Hb 11.8 g/dL with MCV 99 fL, platelet 250,000/mm^3^; serum vitamin B12 level 611 pg/mL, folate level 11.2 ng/mL, and homocysteine level at 11.2 μmol/L. Bilirubin, LDH and haptoglobin were all within the normal range.

### Case 3

A 91 year-old white man with a history of atrial fibrillation, diabetes mellitus type 2 and atherosclerosis presented to the Emergency Department with complaints of increasing fatigue, exertional dyspnea, progressively worsening right upper quadrant pain and mild elevation of serum bilirubin over the past 6 weeks. Mild elevation of total bilirubin (ranging from 1.6 to 1.7 mg/dL) was noticed in a screening blood test three months prior to this presentation and no further work-up was initiated at that time. The patient also had a mild macrocytic anemia (hemoglobin at baseline 11.1 gm/dL with MCV 115 fL). The patient denied hemopytsis, hematochezia, or hematuria. Medications included a glipizide, hydrochlorothiazide, furosemide, and warfarin.

Abnormal physical findings included pale skin, an irregular heart rhythm and right upper quadrant tenderness with slight hepatomegaly, peripheral edema, varicosities and symmetrical weakness in both lower extremities. Thoracic and abdominal computed tomography scans revealed cardiomegaly and pleural effusion. Blood tests revealed WBC 3,100/mm^3^, Hb 6.6 g/dL with MCV 146 fL and platelet 97,000/mm^3^. The serum vitamin B12 level was 162 pg/mL with a normal folate level (16.8 ng/mL) and normal iron study. The homocysteine level was markedly elevated at 129.7 μmol/L, and haptoglobin was < 7 mg/dL. Bone marrow aspirate revealed a cellular bone marrow with 30% nucleated red blood cells with nuclear to cytoplasmic dyssynchrony within the red cell series and dyspoietic changes.

The patient was transfused with 2 units of packed RBC and initiated on intramuscular cobalamin injections, and was discharged in stable condition. Complete blood counts 4 months later revealed WBC 4,400/mm^3^, Hb 11.3 g/dL with MCV 102 fL, and platelet 202,000/mm^3^. Eight months later, vitamin B12 level was 586 pg/mL and his CBC as follows: WBC 5,300, Hb 12.2, and platelet 206,000, which were all within normal ranges. His bilirubin levels were normalized on the follow-up tests after discharge from hospital. Unfortunately, follow up homocysteine levels after cobalamin treatment were not measured and the patient died three years later. However, one of the patient's sons tested positive for homozygous MTHFR C677T mutation.

## Discussion

Concurrent hemolysis in patients with vitamin B12 deficiency is a well-recognized phenomenon. While its mechanisms are not entirely understood, it is believed that the hemolysis results from intramedullary destruction [2]. The patients reported here had ongoing hemolysis as evidenced by the undetectable levels of haptoglobin, with or without elevated LDH and/or bilirubin as well as the presence of schistocytes in the peripheral blood smears (Figures 1, 2). While the third case did not have a follow-up homocysteine level measured, we conjectured that the high homocysteine levels preceding their treatments may have contributed to the patient's hemolysis. The advanced anemia in these three cases that precipitated their hospitalizations correlated well with their high homocysteine levels on admission.

The role of homocysteine in increasing the risk of hemolysis in vitamin B12 and folate deficiency has been demonstrated in vitro [3,4]. However, this phenomenon has not been well appreciated as a possible cause of hemolysis in the clinical setting. We believe that the high homocysteine levels in the above three cases may have played a role in ensuing hemolysis in addition to intramedullary destruction of RBC as evidenced by the fact that both hemolysis and pancytopenia as well as the hyperhomocysteinemia were all corrected by cobalamin treatments. It is also evident that all the three cases reported here either had heterozygous or homozygous MTHFR gene mutations that are known causes of hyperhomocysteinemia though the level of hyperhomocysteinemia may differ between different polymorphisms of MTHFR genes [6]. With the additional impact of vitamin B12 deficiency on the preexisting hyperhomocysteinemia, hemolysis may occur in both intravascular and intramedullary settings, resulting in a drastic reduction of circulating red blood cell mass.

Other evidence implicating the role of homocysteine's hemolytic effects is mainly observed in patients with HELLP (Hemolysis-Elevated Liver Enzymes-Low Platelet) syndrome. While the etiology of HELLP syndrome is not clear, elevated homocysteine levels are often observed among pre-eclamptic patients and have been considered as a risk factor for hemolysis and endothelial damage with ensuing microangiopathy is proposed as the pathophysiologic mechanism of this disorder [7,8]. Other cases in the literature included two young siblings who had a hereditary disorder of cobalamin metabolism (Cbl-C defect) presenting with proteinuria, hematuria, hypertension, and chronic hemolytic anemia with elevated levels of homocysteine [9]. Both patients received renal biopsies with the findings consistent with thrombotic microangiopathy. With the treatment of parenteral hydroxycobalamin and folic acid, the homocysteine levels were reduced significantly with a complete resolution of hemolysis and hematuria [9]. The above two cases and ours suggest the potential association between hyperhomocysteinemia and hemolysis.

Homocysteine has been proposed as a hemolytic toxin [4]. While the exact mechanism of homocysteine's hemolytic effects is not clear, its pro-oxidant attributes have been suggested as a cause [4]. A recent discovery of homocysteine's effect on the down-regulation of cellular glutathione peroxidase-1 activity implicated its role in facilitating the accumulation of reactive oxygen species [10], which may subsequently instigate the oxidative vulnerability of sulfhydryl groups of hemoglobin and thus lead to hemoglobin precipitates within the RBC. However, such intracellular changes would not be expected to lead to the microangiopathic changes in the blood smears seen in our cases.

Another mechanism may be a consequence of the endothelial damage resulting from high homocysteine levels, with ensuing microangiopathy causing hemolysis. Evidence suggesting that hyperhomocysteinemia is associated with thrombosis [11-14] and endothelial damage or dysfunction [5,14] is abundant. In those reports, the pro-oxidant effects of homocyteine were posited as the likely cause of endothelial damage. Studies also suggested that vitamin B12 deficiency may be associated with an increased risk of thrombosis, possibly as a result of hyperhomocysteinemia [14-16]. Furthermore, a recent study demonstrated that endothelial dysfunction can be corrected with vitamin B12 and folic acid treatments in patients with both homozygous MTHFR C677T mutations and vitamin B12 deficiency (with hyperhomocysteinemia) [5]. While some patients with elevated homocysteine levels do not have a complicating hemolytic anemia, as in homocysteinuria and MTHFR gene mutations, the microangiopathic changes in the peripheral blood smears in our cases may be explained by the susceptibility of structurally defective megaloblastic erythrocytes to endothelial damage caused by hyperhomocysteinemia when compared to structurally normal erythrocytes.

Andres *et al. *reported hematological findings in 201 consecutive patients with vitamin B12 deficiency [1]. Approximately 10% of the patients had life threatening hematological manifestations, including symptomatic pancytopenia (5%), "pseudo" thrombotic microangiopathy (2.5%), and hemolytic anemia (1.5%) [1]. A significant proportion of these patients underwent invasive and comprehensive diagnostic panels to rule out other causes of such abnormalities. At times, these patients were misdiagnosed and treated with aggressive measures such as steroids, polyvalent immunoglobulins, and plasmapheresis [1]. It may be possible that the small percentage of cases with evidence of peripheral blood hemolysis were those with extreme elevations of homocysteine levels.

We report here three cases of severe hyperhomocysteinemia caused by vitamin B12 deficiency and MTHFR gene mutations and hemolysis that completely resolved after vitamin B12 therapy. Our cases illustrate the likely culprit of extreme homocysteinemia in precipitating hemolysis in patients with vitamin B12 deficiency and MTHFR gene mutations. Severe hematological complications such as hemolytic anemia and microangiopathy may be more prevalent in patients who have coexisting MTHFR gene mutations and vitamin B12 deficiency possibly related to severely elevated homocysteine levels. To our knowledge, there is no literature report on the association between vitamin B12 deficiency and MTHFR gene mutations as a possible cause of peripheral blood hemolysis. Based upon our observations and the literature review, we are proposing a prospective study to examine the association between vitamin B12 deficiency and MTHFR gene mutations in relationship to the incidence and severity of hyperhomocysteinemia, hemolytic anemia and microangiopathy.

## Consent

Written informed consents have been obtained for two of the three cases. However, neither the patient from the first case or a next of kin could be located despite numerous attempts to obtain consent. We strongly feel that the content within the manuscript is a valuable addition to the scientific literature. We would further expect no objections from the patient or next of kin to the publication since every attempt has been made to maintain the anonymity of the patient.

## Competing interests

The authors declare that they have no competing interests.

## Authors' contributions

The case report was initially originated by UA, JTG, and WC. Later, two more cases were added by WH and PV with UA's coordinating efforts. UA, WH and CTH particularly contributed to the literature update and search. All authors participated in drafting and editing the manuscript. All authors read and approved the final manuscript.

## Authors' information

The authors provided a diverse patient care among different institutions.
